# A mutation-aware proteomics approach for designing a multi-epitope vaccine against multidrug-resistant *Acinetobacter baumannii* and *Pseudomonas aeruginosa*

**DOI:** 10.1128/spectrum.03095-25

**Published:** 2026-02-27

**Authors:** Gul Afshan, Namrah Yaseen, Asad U. Khan

**Affiliations:** 1 Antimicrobial Resistance Lab, Interdisciplinary Biotechnology Unit, Aligarh Muslim University 30037, Aligarh, India; Rowan University Cooper Medical School, Camden, New Jersey, USA

**Keywords:** antimicrobial resistance, bacterial pan-genome analysis, multidrug resistance, extensively drug resistant, vaccine

## Abstract

The increasing prevalence of multidrug-resistant *Acinetobacter baumannii* and *Pseudomonas aeruginosa* in healthcare settings presents a critical challenge to global public health, necessitating the development of effective therapeutic strategies. Here, we report the rationale design of a mutation-aware multi-epitope vaccine targeting conserved, surface-exposed antigens derived from comparative pan-genomic and subtractive proteomic analyses of 30 proteomes. Following stringent antigenicity, allergenicity, toxicity, and homology filtering, three highly antigenic proteins were identified and used to predict immunodominant cytotoxic T-lymphocyte, helper T-lymphocyte, and B-cell epitopes. Epitope selection incorporated mutation probability profiling, and structurally labile residues, particularly serine, were substituted with conservative alternatives such as threonine to enhance conformational stability and HLA binding without compromising immunogenicity. The final construct, comprising 219 amino acids adjuvanted with human β-defensin-3 and connected by cleavable, immunologically inert linkers EAAAK (adjuvant), AAY (CTL), GPGPG (HTL), and KK (B cell) exhibited favorable physicochemical properties, structural stability, and broad global HLA coverage (79%). Structural modeling revealed a high-quality tertiary structure, with 95.8% of residues in favored regions and validated by ProSA and ERRAT scores. Docking with Toll-like receptor 4 demonstrated strong binding affinity supported by molecular dynamics simulations. *In silico* immune simulations predicted strong IgM and IgG responses, sustained memory B- and T-cell populations, and strong cytokine production, including IFN-γ, indicative of skewed Th1 immunity. Codon optimization and cloning analysis confirmed its expression potential in *Escherichia coli*. Collectively, these findings suggest the designed vaccine as a promising therapeutic candidate against polymicrobial infections caused by *A. baumannii* and *P. aeruginosa*, requiring further experimental validation.

**IMPORTANCE:**

Multidrug resistance has become a common phenomenon leading to difficulty in infection control. No options are left to treat such infections after the emergence of colistin resistance. Therefore, this approach to developing multi-epitope therapeutic vaccines is very promising to face the challenge of infection control. This study is based on comparative pan-genomic and subtractive proteomic analyses of 30 proteomes, which provide a wider range of proteins to select multiple epitopes for effective vaccine.

## INTRODUCTION

**Fig 1:**
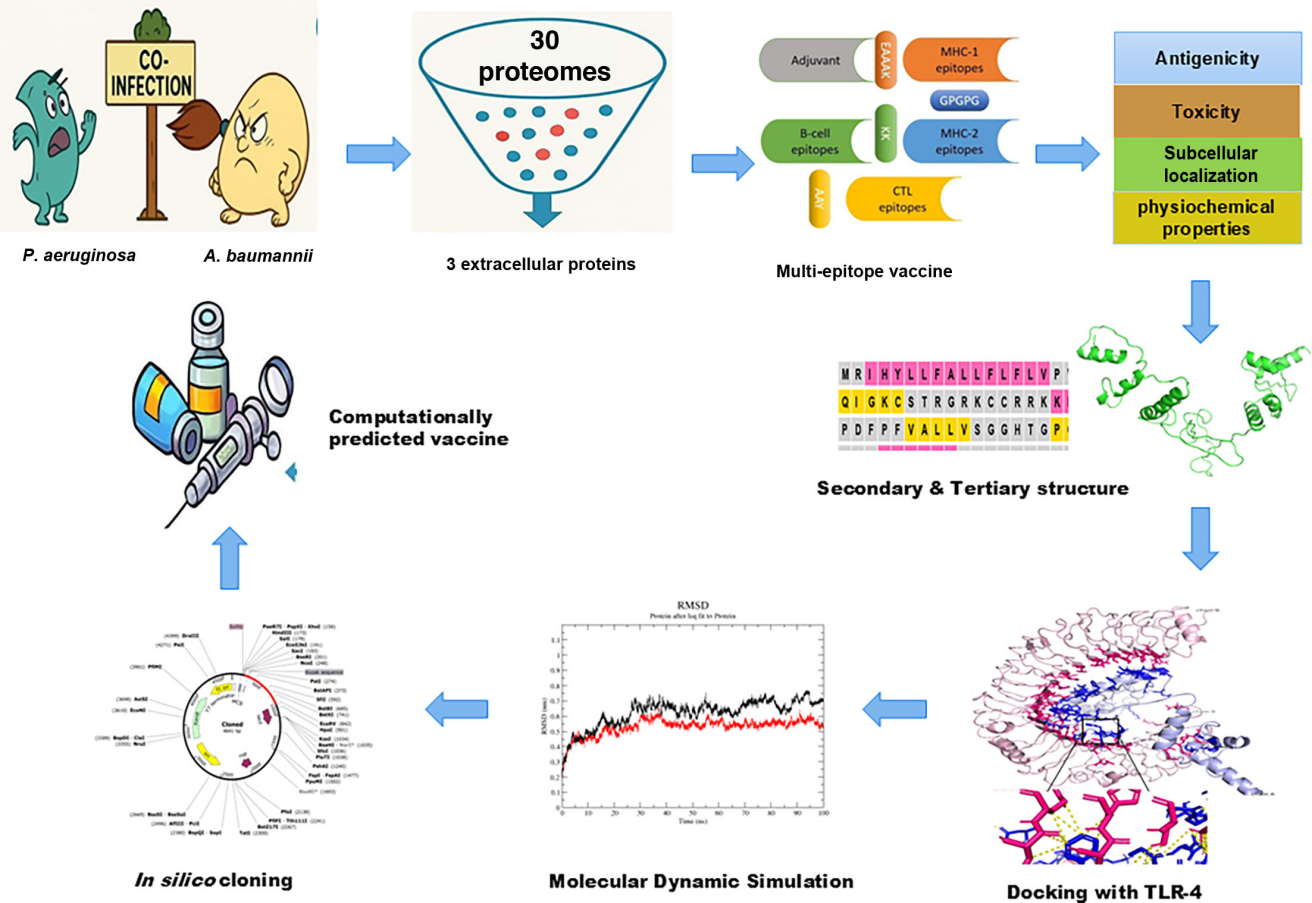
Systematic immunoinformatics-based workflow for the rational design of a multi-epitope vaccine targeting *Pseudomonas aeruginosa* and *Acinetobacter baumannii*.

The global escalation of multidrug-resistant (MDR) bacterial infections has emerged as a pressing public health crisis, further exacerbated by the COVID-19 pandemic, which has led to an increased incidence of secondary bacterial infections in critically ill and ventilated patients (1). Among the most clinically challenging pathogens are *Acinetobacter baumannii* and *Pseudomonas aeruginosa*, two opportunistic gram-negative bacteria frequently implicated in healthcare-associated infections, such as ventilator-associated pneumonia and bloodstream infections. Their exceptional ability to persist on abiotic surfaces and acquire resistance through multiple mechanisms significantly complicates treatment with traditional antibiotics (2). Recent comprehensive reviews have elucidated the complex virulence landscape of *A. baumannii* and *P. aeruginosa*, demonstrating that conserved surface-associated proteins, secretion systems, and regulatory networks play central roles in host colonization, immune evasion, and persistence under antimicrobial pressure (3, 4). In parallel, recent analyses of *Acinetobacter*-directed therapeutic development emphasize the limitations of antibiotic-centric strategies and highlight conserved, immunologically relevant virulence factors as promising alternative targets (5). A retrospective single-center study involving 191 ICU patients (March–May 2020) reported secondary bacterial infections in 29.8% of cases, with *A. baumannii* (28.9%) and *P. aeruginosa* (22.7%) as the predominant pathogens. Notably, 96% of *A. baumannii* isolates exhibited multidrug resistance. Ventilator-associated pneumonia (57.9%) and tracheobronchitis (26.3%) were the most common secondary infections, and co-infected patients experienced significantly prolonged ICU stays (40 vs 17 days), extended mechanical ventilation (24 vs 9 days), and increased mortality (39.7% vs 24.4%; odds ratio: 2.041, 95% CI: 1.080–3.859). These findings reflect a broader global trend observed during the pandemic, which coincided with a marked rise in multidrug-resistant and extensively drug-resistant infections (6). Collectively, this underscores the urgent need for preventive strategies beyond traditional antibiotics. Recent advances in *A. baumannii* vaccine research have demonstrated the feasibility of diverse immunization strategies, including outer membrane protein-based, vesicle-associated, and subunit vaccine platforms, which elicit protective humoral and cellular immune responses in preclinical models (7–9). These studies highlight the importance of targeting conserved, surface-exposed antigens to overcome strain heterogeneity and immune evasion. Notably, the recombinant hybrid vaccine IC43, comprising OprF and OprI, reached phase II/phase III clinical trials (10) but failed to achieve statistically significant protection, highlighting the limitations of conventional vaccine approaches (11). A similar lack of translational success has been observed in the context of *A. baumannii*, where despite the identification of promising antigenic targets, no candidate has yet advanced to clinical trial (12). These outcomes point to the critical need for rational, multi-parametric vaccine design approaches that prioritize immunogenic stability, antigenic conservation, and broad-spectrum coverage (13). To address these challenges, we employed a mutation-aware (14), proteomics-guided computational framework (15) to design a multi-epitope subunit vaccine effective against both *A. baumannii* and *P. aeruginosa*. This approach began with a comprehensive pan-genome analysis of 15 proteomes from each species to identify conserved, extracellular, and non-homologous proteins with high antigenic potential. These prioritized proteins served as the basis for cytotoxic T-lymphocyte (CTL), helper T-lymphocyte (HTL), and B-cell epitope prediction. The shortlisted epitopes were rigorously evaluated using a multi-criteria screening process that considered immunogenicity, antigenicity, allergenicity, toxicity, and hydrophilicity. A distinctive aspect of our methodology involved the integration of mutation probability profiling to improve epitope stability against evolutionary pressure. Specifically, we analyzed anchor residues within predicted epitopes for susceptibility to amino acid substitutions, focusing particularly on serine residues frequently located at mutable sites. Where appropriate, conservative substitutions, such as serine to threonine, were introduced to enhance major histocompatibility complex (MHC) binding affinity and structural resilience without compromising immunological relevance. This mutation-aware optimization was intended to mitigate immune escape and enhance the long-term efficacy of our vaccine construct. The final multi-epitope vaccine (MEV) was designed by linking the selected HTL, B-cell, and CTL epitopes using flexible linkers and fusing them with the human β-defensin-3 adjuvant (16) to potentiate immune responses. The physicochemical and immunological properties of the chimeric construct were comprehensively evaluated through tertiary structure prediction and modeling, immune simulations, molecular docking with innate immune receptors, and molecular dynamics (MD) simulation. The sequence was reverse translated and codon optimized to be further cloned into the pET28a(+) expression vector for translational feasibility in *Escherichia coli*. Figure 1 presents a comprehensive schematic of the integrative immunoinformatics-driven workflow utilized for the rational design of a multi-epitope vaccine candidate targeting co-infections caused by *P. aeruginosa* and *A. baumannii*. Collectively, this study presents a potent and integrative *in silico* strategy that couples comparative proteomics, immunoinformatics, and evolutionary modeling to advance next-generation vaccine design against high-priority MDR pathogens *A. baumannii* and *P. aeruginosa*.

## MATERIALS AND METHODS

### Core proteome identification and subtractive proteomics

Thirty proteomes (15 each of *P. aeruginosa* and *A. baumannii*), both classified as WHO critical priority pathogens, were retrieved from the NCBI database, and pan-genome analysis was performed via the Bacterial Pan Genome Analysis Tool (BPGA) (17). Core proteins conserved across all strains were identified as potential broad-spectrum targets. Non-human homologs were selected through BLASTp (18) against the human proteome (e-value ≤1e−4, identity ≤30%, coverage ≥70%) to avoid host cross-reactivity. Subcellular localization was predicted by PSORTb 3.0 (19) to prioritize extracellular proteins as vaccine targets. Antigenicity was evaluated using the VaxiJen server (20) (threshold >0.4), and allergenicity was assessed via AllerTOP v2.0 (21) to ensure immunogenicity and safety of the selected candidates.

### Epitope prediction and selection

CTL, HTL, and linear B-cell (LBL) epitopes were predicted from the filtered proteins. CTL and HTL epitopes were anticipated using the MHC-I (22) and MHC-II binding prediction (23) tools, respectively, available at the Immune Epitope Database (IEDB) (24), employing the consensus method on input protein sequences in FASTA format. The human host was specified as the source of all MHC alleles. Epitopes with predicted binding affinities (IC_50_ < 500 nM) were selected for their strong binding potential (25). Linear B-cell epitopes were predicted using the BepiPred 2.0 server (26). The antigenicity, allergenicity, and toxicity of the shortlisted epitopes were subsequently assessed using VaxiJen v2.0 (20), AllerTOP v.2 (21), and ToxinPred servers (27), respectively. In parallel, the selected epitopes were screened for the presence of position-specific serine residues, and site-directed mutagenesis was performed to assess their structural impact. The energy profiles of the native and mutated epitopes were evaluated using Aggrescan3D (28) to predict aggregation propensity and stability. Furthermore, the antigenicity and binding affinity of the mutated epitopes (23) were thoroughly assessed to validate their potential for inclusion in the vaccine formulation.

### Population coverage analysis

The heterogeneity of ethnic and regional populations significantly influences the distribution and expression of human leukocyte antigen (HLA) alleles, which in turn affects vaccine efficacy. To evaluate the breadth of immune coverage conferred by the selected epitopes, population coverage analysis was performed using the IEDB Population Coverage Tool (29). This computational tool integrates HLA-binding data corresponding to the identified MHC class I and class II epitopes and estimates the prospective population coverage by analyzing the global distribution of HLA alleles. The analysis provides a quantitative assessment of the extent to which each epitope is likely to elicit an immune response across diverse human populations, thereby informing the design of broadly protective immunogens.

### Vaccine construction and physicochemical properties

The multi-epitope vaccine construct was rationally designed by integrating CTL, HTL, and LBL epitopes, in conjunction with relevant adjuvants and linkers to enhance immunogenicity and structural stability. Human β-defensin 3, retrieved from UniProt (UniProt ID Q5U7J2) (30, 31), was selected as the immunostimulatory adjuvant due to its potent ability to activate the innate immune response. The adjuvant was fused to the N-terminal of the sequence using an EAAAK linker, which confers structural rigidity and functional separation between domains. Subsequently, AAY, GPGPG, and KK linkers were employed to join the CTL, HTL, and LBL epitopes, respectively (32). These linkers were selected based on their proven efficacy in preserving epitope immunogenicity, enhancing epitope processing, and ensuring proper spatial configuration within the chimeric vaccine sequence. The physicochemical properties of the MEV construct were performed using a suite of computational tools. Initially, key physicochemical parameters including theoretical isoelectric point (pI), molecular weight, instability index, grand average of hydropathicity (GRAVY), aliphatic index, and estimated *in vivo* and *in vitro* half-lives were computed using the ExPASy ProtParam server (33). Furthermore, the antigenicity and allergenicity of the MEV construct were assessed via the VaxiJen and AllerTOP, respectively, to evaluate the potential risk of hypersensitivity reactions.

### Structural analysis and refinement

The secondary structure composition comprising α-helices, β-turns, and random coils was estimated by the PSIPRED workbench (34). For tertiary structure modeling, the Sratch suite 3DPro server (35) was employed to generate the initial three-dimensional structure of the MEV construct, which underwent further refinement through the GalaxyRefine server (36) to enhance structural quality and stability. Structural validation was conducted using the PROCHECK server (37) to analyze Ramachandran plot statistics and assess stereochemical quality. Additionally, ProSA-web (38) and ERRAT server (39) were used to identify potential structural errors and to assess the structural integrity and reliability of the predicted 3D model. Finally, both conformational and linear B-cell epitopes were identified by the ElliPro tool (40) to assess surface accessibility and potential for antibody recognition.

### Molecular docking of the vaccine construct with Toll-like receptor 4

The three-dimensional structure of human Toll-like receptor 4 (TLR4, PDB ID: 3FXI) (41) was retrieved from the Protein Data Bank (42). All ligands and water molecules were removed from the structure prior to molecular docking to eliminate potential interference. The CASTp server (43) was used to characterize the interaction interface and key residues involved in protein–protein interactions. The refined multi-epitope vaccine construct and the processed TLR4 structure were docked using the HADDOCK 2.4 server (44), which performs information-driven flexible docking based on predicted interaction sites. The resulting docked complex was analyzed using the PDBsum server (45) to examine interfacial residues and characterize interactions such as hydrogen bonds, salt bridges, and non-bonded contacts. To evaluate the binding affinity of the docked complex, the top-ranked model was analyzed using the PRODIGY server (46) at 37°C. Binding affinity was reported as the dissociation constant (Kd), and the binding free energy (ΔG) was calculated in kilocalories per mole, indicating the thermodynamic stability between the vaccine construct and TLR4 (47).

### MD simulation

MD simulations were performed using GROMACS to investigate the stability and interaction dynamics of our vaccine construct with receptor TLR4. The OPLS_2005 force field was employed for parameterization (48). The system was enclosed within a cubic simulation box, ensuring at least 1 nm of solvent padding around the solute, and solvated with explicit water molecules. To mimic physiological conditions, chloride and sodium ions were added to neutralize the system, followed by energy minimization using the steepest descent algorithm for 50,000 steps. The system was then equilibrated under constant volume (NVT) and constant pressure (NPT) ensembles, maintaining a temperature of 300 K and a pressure of 1 bar (49). The production run was executed for 100 ns using the particle mesh Ewald method for long-range electrostatic interactions. Trajectory frames were recorded at 1 ns intervals throughout the simulation. The conformational stability and deviation were measured using root mean square deviation (RMSD) and root mean square fluctuation (RMSF) (50).

### Codon optimization and *in silico* cloning

Codon optimization is essential for enhancing the expression efficiency of heterologous genes in a specific host by aligning codon usage with the host’s translational machinery. The protein sequence was reverse translated by the EMBOSS Backtranseq tool (51), and codon optimization of the vaccine construct was evaluated using the JCat platform (52) to suit the *E. coli* expression system. The optimized nucleotide sequence was then inserted *in silico* into the pET28a(+) expression vector (53), strategically inserted between the Eco53kI and EcoRV restriction sites using the SnapGene tool (54).

### Immune simulation

The C-ImmSim server (55) was used to potentiate the immune response and immunogenicity of the designed vaccine construct. Based on the standard 1-month interval between doses, simulations were conducted at time steps 1, 84, and 168, corresponding to three vaccine administrations (with each time step representing 8 hours) (56). All other simulation parameters were kept at default settings.

## RESULTS

### Core proteome identification and subtractive proteomics

The complete proteomes of 30 clinically relevant multidrug-resistant bacterial strains, 15 each of *P. aeruginosa* and *A. baumannii*, were retrieved from the UniProt database (Table 1). A comprehensive subtractive proteomics approach was employed, integrated with the BPG pipeline, to identify conserved and immunologically relevant targets for vaccine design. Initial pan-genome analysis yielded 4,503 conserved proteins across the selected strains. Subcellular localization prediction using PSORTb identified 54 of these as extracellular, rendering them accessible to host immune surveillance. Subsequent antigenicity assessment revealed 45 proteins with high antigenic potential. These candidates were then subjected to homology screening against the human proteome using BLASTp to eliminate self-antigens, resulting in the identification of 3 non-homologous, extracellular, and highly antigenic proteins. Toxicity prediction confirmed their non-toxic nature (Table 2). These three proteins with protein IDs >core/5079/16/Org16_Gene62, >core/5079/24/Org24_Gene362, and >core/5079/8/Org8_Gene519, respectively, represent promising targets for the development of a broad-spectrum, multi-epitope vaccine aimed at eliciting protective immune responses against MDR *A. baumannii* and *P. aeruginosa*.

**TABLE 1:** Selected *Acinetobacter baumannii* and *Pseudomonas aeruginosa* strains with their UniProt proteome IDs, strain identifiers, and gene counts

Proteome ID	Strain ID	Gene count	Organism
UP000005740	ATCC19606	3,765	*A. baumannii*
UP000031035	ATCC17945	3,626	*A. baumannii*
UP000032746	AB5075-UW	3,839	*A. baumannii*
UP000050896	ABBL070	3,590	*A. baumannii*
UP000051023	ABBL067b	3,665	*A. baumannii*
UP000072389	ATCC17978	3,661	*A. baumannii*
UP000179937	XH647	3,802	*A. baumannii*
UP000194553	ARLG1317	4,042	*A. baumannii*
UP000237823	KUFAR57	3,719	*A. baumannii*
UP000248662	R10	3,568	*A. baumannii*
UP000197394	AB360	3,920	*A. baumannii*
UP000249678	NCTC13421	3,735	*A. baumannii*
UP000252290	4300STDY7045845	4,009	*A. baumannii*
UP000252694	4300STDY7045823	4,058	*A. baumannii*
UP000260332	4300STDY7045712	3,928	*A. baumannii*
UP000005606	2_1_26	6,048	*P. aeruginosa*
UP000027768	MRSN18971	5,704	*P. aeruginosa*
UP000027947	KIB	4,835	*P. aeruginosa*
UP000028369	JMM	5,842	*P. aeruginosa*
UP000029102	PAG	6,273	*P. aeruginosa*
UP000029588	ATCC33988	5,830	*P. aeruginosa*
UP000045039	P19	6,934	*P. aeruginosa*
UP000253594	B2-305	8,711	*P. aeruginosa*
UP000276985	MRSN6241	6,717	*P. aeruginosa*
UP000284767	PA-W36	6,629	*P. aeruginosa*
UP000284878	CPHL10662	6,459	*P. aeruginosa*
UP000344659	NCTC13621	7,197	*P. aeruginosa*
UP000433532	PA221	6,646	*P. aeruginosa*
UP000644192	VNMU148	7,390	*P. aeruginosa*
UP000712497	NMI2426/06	6,567	*P. aeruginosa*

**TABLE 2:** List of selected proteins with their protein IDs, along with *in silico* predictions of antigenic potential, allergenic propensity, toxicity, and subcellular localization

Protein ID	Antigenicity	Allergenicity	Toxicity	Subcellular location
>core/5079/16/Org16_Gene62	0.5157	Non-allergen	Non-toxic	Extracellular
>core/5079/24/Org24_Gene362	0.4997	Non-allergen	Non-toxic	Extracellular
>core/5079/8/Org8_Gene519	0.509	Non-allergen	Non-toxic	Extracellular

### Identification and selection of putative epitopes

A total of 12 CTL epitopes (9-mer) were predicted from the three prioritized target proteins using MHC class I binding prediction tools. Among these, only one epitope, MTDRPGLDF (protein ID: >core/5079/24/Org24_Gene362, antigenicity:1.4761) fulfilled the essential criteria of high antigenicity, non-allergenicity, and non-toxicity (Table 3). Concurrently, MHC class II binding analysis yielded 17 unique HTL epitopes (15-mer). Of these, three HTL epitopes—LDFSFSGLKTAVSVQ (protein ID: >core/5079/16/Org16_Gene62, antigenicity:0.999), PEFPFVALLVSGGHT (protein ID: >core/5079/8/Org8_Gene519; antigenicity:0.8943), and GGVSANQALRGGLEK (protein ID: >core/5079/24/Org24_Gene362, antigenicity: 1.0117)—exhibited strong antigenic potential (Table 4). In parallel, MHC class I analysis also predicted 32 distinct HTL epitopes (9-mer), of which KTAVSVQLK (protein ID: >core/5079/16/Org16_Gene62, antigenicity: 1.2874), EEQPPRFPF (protein ID: >core/5079/24/Org24_Gene362, antigenicity: 1.2706), and PEFPFVALL (protein ID: >core/5079/8/Org8_Gene519, antigenicity: 1.2293) met all selection criteria; among them, one epitope (PEFPFVALL) was predicted to induce interleukin-4, further supporting its role in humoral immune stimulation (Table 5). Additionally, 10 LBL epitopes were identified, and following evaluation for immunogenicity, allergenicity, and toxicity, three epitopes—ALSGDPLAFEFPRPMLHQGLD (protein ID: >core/5079/16/Org16_Gene62, antigenicity: 0.634), QRCVEAGDDSEQT (protein ID: >core/5079/24/Org24_Gene362, antigenicity: 1.4317), and AGQHDGLAVTTT (protein ID: >core/5079/8/Org8_Gene519, antigenicity: 1.0511)—were shortlisted for inclusion in the final vaccine construct (Table 6). Furthermore, residue-level conformational stability analysis via the Aggrescan3D server identified two serine-containing epitopes exhibiting favorable energetic profiles upon *in silico* substitution of serine with threonine, indicative of enhanced structural integrity (Table 7). The remaining epitopes did not demonstrate appreciable ΔG improvements and were therefore retained in their native conformations. Subsequent HLA-binding affinity assessments of the mutated epitopes revealed increased complex stability with their corresponding HLA alleles, supporting their incorporation in the final multi-epitope vaccine sequence.

**TABLE 3:** Predicted CTL epitopes with positions, length, and antigenicity scores

Epitopes	Protein ID	Start	End	Antigenicity
MTDRPGLDF	>core/5079/24/Org24_Gene362	201	209	1.4761

**TABLE 4:** Predicted MHC-II epitopes with positions, alleles, and antigenicity scores

Epitopes	Protein ID	Start	End	Allele	Antigenicity
LDFSFSGLKTAVSVQ	>core/5079/16/Org16_Gene62	206	220	DRB1_0408 HLA-DPA1*01:03/DPB1*02:01 HLA-DRB1*01:01 HLA-DRB1*11:01 HLA-DRB5*01:01 HLA-DRB1*09:01 HLA-DQA1*01:02/DQB1*06:02 HLA-DRB1*04:01 HLA-DRB1*04:05 HLA-DRB1*08:02 HLA-DRB1*07:01 HLA-DPA1*03:01/DPB1*04:02 HLA-DQA1*05:01/DQB1*03:01 HLA-DPA1*01:03/DPB1*02:01 HLA-DRB1*15:01 HLA-DPA1*02:01/DPB1*01:01 HLA-DRB4*01:01 HLA-DPA1*02:01/DPB1*14:01 HLA-DQA1*04:01/DQB1*04:02 HLA-DRB1*13:02 HLA-DRB1*12:01 HLA-DPA1*02:01/DPB1*05:01 HLA-DQA1*03:01/DQB1*03:02 HLA-DPA1*01:03/DPB1*04:01 HLA-DRB1*03:01 HLA-DRB3*02:02 HLA-DQA1*05:01/DQB1*02:01 HLA-DRB3*01:01 HLA-DQA1*01:01/DQB1*05:01	0.999
PEFPFVALLVSGGHT	>core/5079/8/Org8_Gene519	126	140	DRB1_1001 HLA-DRB1*07:01 HLA-DQA1*05:01/DQB1*03:01 HLA-DRB1*08:02 HLA-DRB4*01:01 HLA-DRB1*01:01 HLA-DRB1*04:05 HLA-DRB5*01:01 HLA-DRB1*09:01 HLA-DRB1*11:01 HLA-DRB1*15:01 HLA-DRB1*04:01 HLA-DPA1*01:03/DPB1*02:01 HLA-DPA1*03:01/DPB1*04:02 HLA-DPA1*02:01/DPB1*01:01 HLA-DRB1*12:01 HLA-DQA1*01:02/DQB1*06:02 HLA-DPA1*01:03/DPB1*04:01 HLA-DQA1*04:01/DQB1*04:02 HLA-DPA1*02:01/DPB1*05:01 HLA-DQA1*05:01/DQB1*02:01 HLA-DPA1*02:01/DPB1*14:01 HLA-DRB1*13:02 HLA-DQA1*03:01/DQB1*03:02 HLA-DRB3*01:01 HLA-DQA1*01:01/DQB1*05:01 HLA-DRB3*02:02 HLA-DRB1*03:01	0.8943
GGVSANQALRGGLEK	>core/5079/24/Org24_Gene362	271	285	DRB3_0202 HLA-DQA1*01:02/DQB1*06:02 HLA-DRB1*03:01 HLA-DRB1*01:01 HLA-DRB1*13:02 HLA-DRB3*02:02 HLA-DRB1*01:01 HLA-DRB1*03:01 HLA-DQA1*01:02/DQB1*06:02 HLA-DQA1*05:01/DQB1*03:01 HLA-DRB1*09:01 HLA-DRB4*01:01 HLA-DRB5*01:01 HLA-DRB1*11:01 HLA-DRB1*04:01 HLA-DRB1*07:01 HLA-DRB1*15:01 HLA-DRB1*08:02 HLA-DRB1*12:01 HLA-DQA1*03:01/DQB1*03:02 HLA-DQA1*04:01/DQB1*04:02 HLA-DRB1*04:05 HLA-DQA1*05:01/DQB1*02:01 HLA-DPA1*02:01/DPB1*01:01 HLA-DPA1*02:01/DPB1*14:01 HLA-DRB3*01:01 HLA-DPA1*02:01/DPB1*05:01 HLA-DPA1*01:03/DPB1*02:01 HLA-DPA1*01:03/DPB1*04:01 HLA-DPA1*03:01/DPB1*04:02 HLA-DQA1*01:01/DQB1*05:01	1.0117

**TABLE 5:** Predicted MHC-I epitopes with positions, alleles, and antigenicity scores

Epitopes	Protein ID	Start	End	MHC-1 alleles	Antigenicity	IL-4^*a*^ inducer
KTAVSVQLK	>core/5079/16/Org16_Gene62	214	222	HLA-A*03:01 HLA-A*68:01 HLA-A*31:01 HLA-A*01:01 HLA-A*02:01 HLA-A*24:02 HLA-A*26:01 HLA-B*07:02 HLA-B*27:05 HLA-B*39:01 HLA-B*40:01 HLA-B*15:01	1.2874	Non-inducer
EEQPPRFPF	>core/5079/24/Org24_Gene362	122	130	HLA-B*40:01 HLA-B*44:02 HLA-A*02:01 HLA-A*01:01 HLA-A*03:01 HLA-A*24:02 HLA-A*26:01 HLA-B*07:02 HLA-B*08:01 HLA-B*39:01 HLA-B*40:01 HLA-B*58:01 HLA-B*15:01	1.2706	Non-inducer
PEFPFVALL	>core/5079/8/Org8_Gene519	126	134	HLA-B*40:01 HLA-A*01:01 HLA-A*03:01 HLA-A*02:01 HLA-A*24:02 HLA-A*26:01	1.2293	IL4 inducer

^*a*^ IL-4, interleukin-4.

**TABLE 6:** Predicted B-cell epitopes with positions and antigenicity scores

Epitopes	Protein ID	Start	End	Antigenicity
ALSGDPLAFEFPRPMLHQGLD	>core/5079/16/Org16_Gene62	187	207	0.634
QRCVEAGDDSEQT	>core/5079/24/Org24_Gene362	223	235	1.4317
AGQHDGLAVTTT	>core/5079/8/Org8_Gene519	313	324	1.0511

**TABLE 7:** Comparative analysis of original and mutated epitopes, including start and end positions within the protein sequence, predicted antigenicity scores, and IC_50_ values, highlighting the effect of sequence modifications on epitope binding affinity and immunogenic potential

Epitopes	Start	End	Antigenicity	IC_50_
Original epitopes				
PEFPFVALLVSGGHT	126	140	0.8943	95.8
GGVSANQALRGGLEK	271	285	1.0117	80.2
Mutated epitopes				
PEFPFVALLVTGGHT	126	140	0.9694	76
GGVTANQALRGGLEK	271	285	0.9918	58.2

### Population coverage

**Fig 2:**
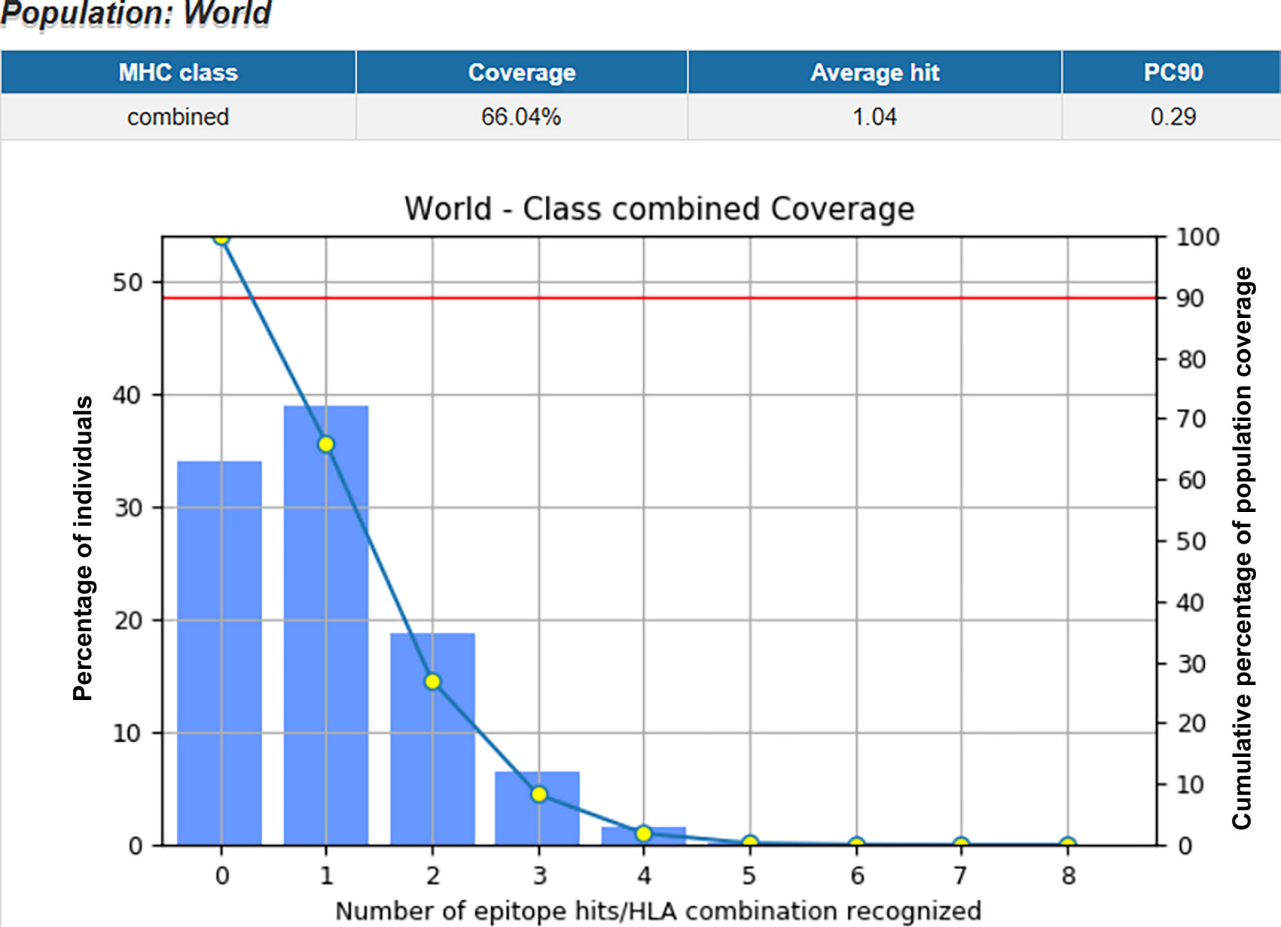
Worldwide distribution of population coverage for the combined HLA classes, achieving an estimated 79% global coverage.

The multi-epitope vaccine construct was evaluated for its potential to elicit cellular immune responses across diverse human populations using the IEDB Population Coverage tool. T-cell epitope predictions were performed against a comprehensive set of 54 HLA class I and II alleles. The analysis revealed a projected global population coverage of approximately 79%, with a PC90 value of 0.47, indicating that 90% of the population would recognize at least one epitope with an average of 0.47 HLA hits per individual (Fig. 2). Collectively, the data predict that the vaccine is likely to promote broad-spectrum T-cell-mediated immunity in a global population context.

### Vaccine construction and physicochemical properties

**Fig 3:**
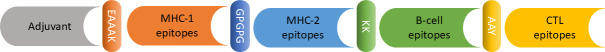
Schematic illustration of the multi-epitope vaccine design incorporating MHC-I, MHC-II, B-cell, and CTL epitopes. The β-defensin adjuvant is fused using an EAAAK linker (gray); MHC-I epitopes are connected through GPGPG linkers (orange), MHC-II epitopes through GPGPG linkers (blue), B-cell epitopes via KK linkers (green), and CTL epitopes via AAY linkers (yellow).

The MEV construct was designed based on stringent immunological and physicochemical parameters, ensuring high antigenicity and immunogenicity while eliminating allergenic and toxic components. The construct integrates one CTL, six HTL, and three B-cell epitopes, linked via immunologically relevant spacers AAY, GPGPG, and KK and fused at the N-terminus with human β-defensin using an EAAAK linker for adjuvant activity and structural stability (Fig. 3). The final construct comprises 219 amino acid residues, has a molecular weight of 23,285.96 Da and a theoretical pI of 9.55. It exhibits a predicted half-life of 30 hours (mammalian reticulocytes, *in vitro*), >20 hours (yeast, *in vivo*), and >10 hours (*E. coli*, *in vivo*). An instability index of 36.30 classifies it as a stable protein, while the aliphatic index (71.28) and the GRAVY score (−0.306) (Table 8) suggest moderate thermostability and a hydrophilic nature.

**TABLE 8:** Predicted physicochemical and immunological properties of the designed vaccine construct

EXPASY parameter	Value
Amino acids	219
Molecular weight	23,285.96 Da
Theoretical pI	9.55
Estimated half-life	>10 hours (*Escherichia coli*, *in vivo*)
Instability index	36.30
Aliphatic index	71.28
Grand average of hydropathicity	−0.306
Solubility (Solpro)	0.950
Antigenicity (Vaxijen)	1.1706

### Structural prediction and validation

**Fig 4:**
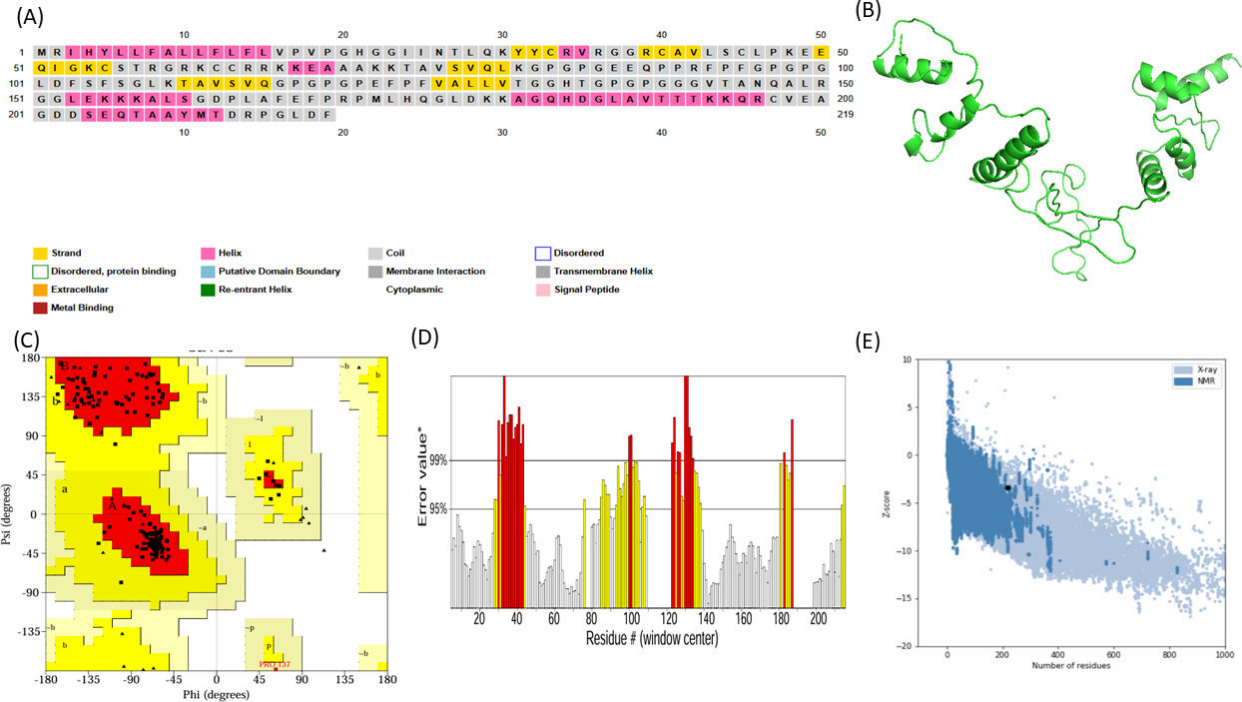
(**A**) Predicted secondary structure profile of the designed vaccine construct. (**B**) Three-dimensional structural model of the vaccine. (**C**) Ramachandran plot illustrating the conformational distribution of amino acid residues, with red regions representing α-helices and yellow regions representing β-sheets. (**D**) ERRAT quality factor assessment of the modeled structure. (**E**) ProSA-web *Z*-score of the vaccine construct (black dot) indicating overall structural reliability.

**Fig 5:**
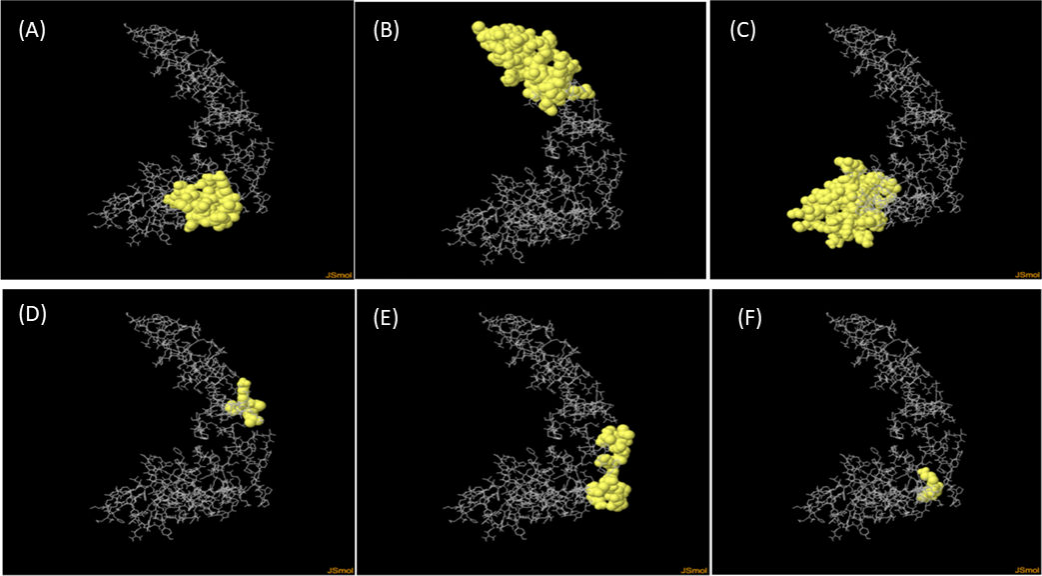
The conformational B-cell epitopes in a three-dimensional model of the vaccine: (**A**) epitope with 17 residues, (**B**) epitope with 45 residues, (**C**) epitope with 45 residues, (**D**) epitope with 5 residues, (**E**) epitope with 14 residues, and (**F**) epitope with 3 residues.

The structural characteristics of the MEV construct were analyzed to ensure its suitability for downstream applications. Secondary structure prediction using PSIPRED revealed 23.28% α-helical residues, 12.78% extended strands, and 63.93% random coils (Fig. 4A). Tertiary structure modeling was performed using Robetta and 3Dpro, with the top-ranked model selected for refinement via GalaxyRefine (Fig. 4B). Structural validation identified 95.8% of residues in favored regions, and only 4.2% in allowed regions on the Ramachandran plot (Fig. 4C). The refined model exhibited a quality score of 65.93 (Fig. 4D) and a *Z*-score of −3.41 (Fig. 4E), confirming acceptable stereochemical integrity and structural reliability for further molecular docking and immunoinformatics analyses. ElliPro server identified six conformational B-cell epitopes (Fig. 5 and Table 9) and seven linear B-cell epitopes (Table 10).

**TABLE 9:** Conformational B-cell epitopes of the designed vaccine construct predicted using the ElliPro server, detailing epitope residue positions, number of residues, and associated prediction scores

No.	Residues	No. of residues	Score
1	_:S77, _:V78, _:Q79, _:L80, _:K81, _:G82, _:P83, _:G84, _:P85, _:G86, _:E87, _:E88, _:Q89, _:P90, _:P91, _:R92, _:S104	17	0.736
2	_:E167, _:F168, _:P169, _:Q175, _:G176, _:L177, _:D178, _:K179, _:K180, _:A181, _:G182, _:Q183, _:H184, _:A188, _:V189, _:T190, _:T191, _:T192, _:K193, _:K194, _:Q195, _:R196, _:C197, _:V198, _:E199, _:A200, _:G201, _:D202, _:D203, _:S204, _:E205, _:Q206, _:T207, _:A208, _:A209, _:Y210, _:M211, _:T212, _:D213, _:R214, _:P215, _:G216, _:L217, _:D218, _:F219	45	0.701
3	_:M1, _:R2, _:I3, _:H4, _:Y5, _:L6, _:L7, _:F8, _:A9, _:L10, _:L11, _:F12, _:L13, _:F14, _:L15, _:V16, _:V18, _:P19, _:G20, _:G22, _:G23, _:I24, _:I25, _:N26, _:T27, _:L28, _:Q29, _:K30, _:Y31, _:Y32, _:C33, _:R34, _:V35, _:R36, _:G37, _:G38, _:R39, _:A41, _:V42, _:L43, _:E50, _:I52, _:G53, _:K54, _:C55	45	0.68
4	_:K157, _:A158, _:L159, _:S160, _:G161	5	0.603
5	_:K73, _:T110, _:A111, _:V112, _:S113, _:V114, _:Q115, _:G116, _:P117, _:G118, _:P119, _:G120, _:P121, _:E122	14	0.598
6	_:A70, _:A71, _:K72	3	0.502

**TABLE 10:** Linear B-cell epitopes predicted by ElliPro webserver^*a*^

No.	Chain	Start	End	Peptide	No. of residues	Score
1	–	190	219	TTTKKQRCVEAGDDSEQTAAYMTDRPGLDF	30	0.8
2	–	78	92	VQLKGPGPGEEQPPR	15	0.779
3	–	18	40	VPGHGGIINTLQKYYCRVRGGRC	23	0.625
4	–	111	124	AVSVQGPGPGPEFP	14	0.615
5	–	157	161	KALSG	5	0.603
6	–	174	183	HQGLDKKAGQ	10	0.573
7	–	52	55	IGKC	4	0.519

^*a*^ – indicates single chain, with no differentiation in chain A and B.

### Molecular docking with TLR4

**Fig 6:**
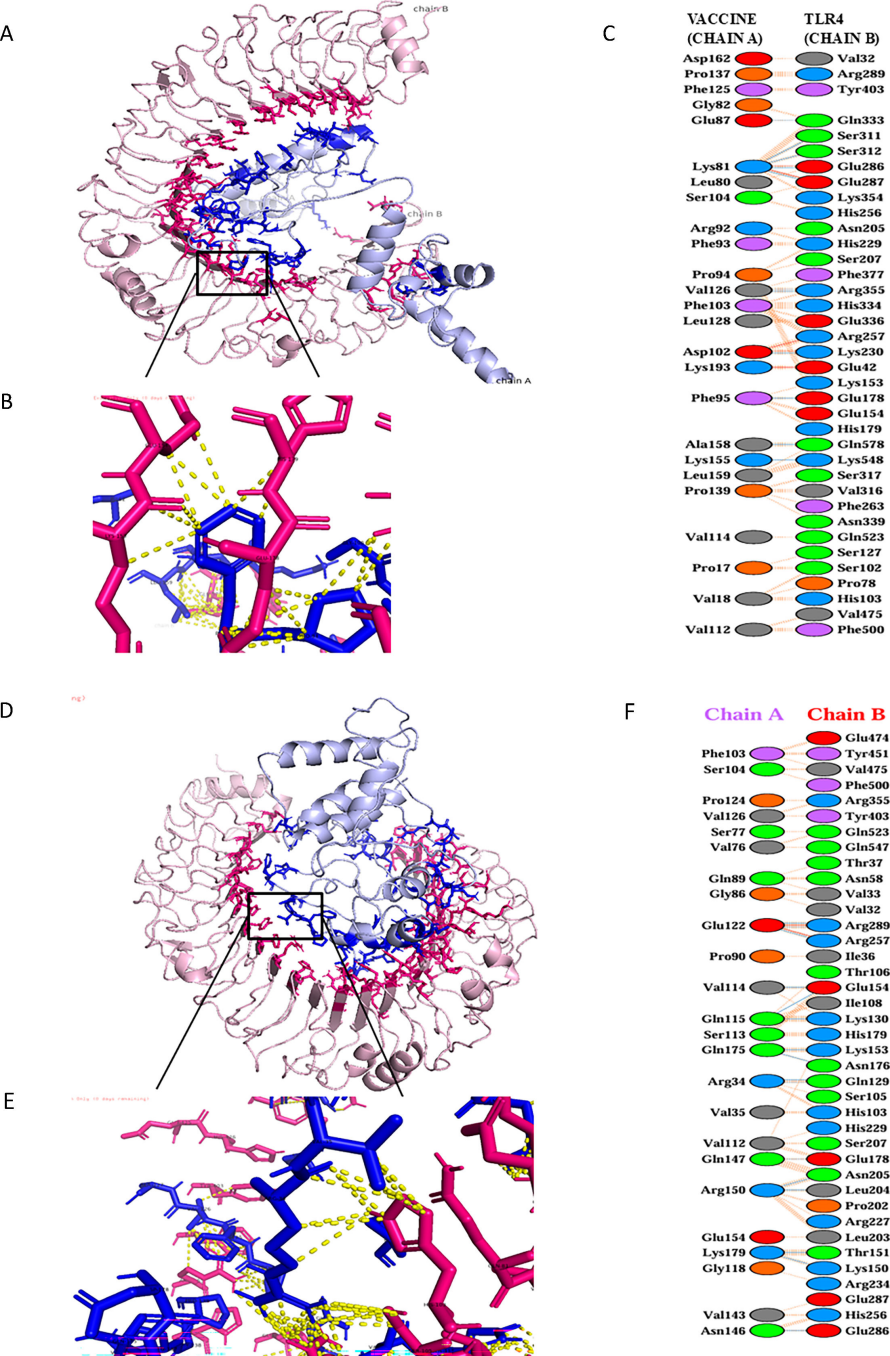
(**A**) Docking model of the vaccine–TLR4 complex, with the vaccine in blue and TLR4 in pink. (**B**) Close-up of interacting residues, shown as sticks with chain IDs and residue numbers; interactions are depicted with colored dashed lines. (**C**) Summary of interacting residues and bond types between the vaccine and TLR4 chains. (**D**) Docking model of the original vaccine–TLR4 complex, with the vaccine in blue and TLR4 in pink. (**E**) Close-up of interacting residues, shown as sticks with chain IDs and residue numbers; interactions are depicted with colored dashed lines. (**F**) Summary of interacting residues and bond types between the vaccine (chain A) and the TLR4 chain (chain B).

TLR4, a key innate immune receptor, was selected for docking due to its role in recognizing pathogen-associated molecular patterns. The crystal structure of human TLR4 was processed and optimized via UCSF Chimera, retaining chain A and removing other chains. Active binding sites were predicted using the CASTp server. Protein–protein docking of the MEV construct and TLR4 was performed using HADDOCK v2.4, with the best cluster selected based on the lowest HADDOCK score. The docked complex revealed strong interaction, forming 5 salt bridges, 11 hydrogen bonds, and 146 non-bonded contacts (Fig. 6A and B). Interface analysis via PDBsum indicated 25 MEV residues interacting with 37 TLR4 residues, with interface areas of 1,683 and 1,458 Å², respectively. Binding affinity predicted by PRODIGY showed a ΔG of −16.4 kcal/mol with a dissociation constant (Kd) of 8.7 × 10^−13^ M (Table 11), indicating a highly stable and energetically favorable interaction. These results highlight the strong binding stability of the mutated MEV–TLR4 complex, comparable to that of the original protein (ΔG = −13.4 kcal/mol, Kd = 1.4 × 10^−10^ M) (Table 11), and support the structural integrity and effectiveness of the engineered vaccine in engaging innate immune receptors.

**TABLE 11:** Comparative docking and binding parameters of the original and mutated vaccine–TLR4 complexes^*a*^

Parameter	Original vaccine-TLR4 complex	Mutated vaccine-TLR4 complex
HADDOCK score (kJ mol^−1^)	11.6 ± 10.9	−26.1 ± 7.4
Cluster size	38	6
RMSD from the overall lowest energy structure (nm)	0.8 ± 0.4	1.4 ± 0.8
van der Waals energy (kJ mol^−1^)	−78.6 ± 6.0	−71.5 ± 9.6
Electrostatic energy (kJ mol^−1^)	−257.2 ± 40.1	−443.1 ± 76.9
Desolvation energy (kJ mol^−1^)	−5.1 ± 4.5	−37.6 ± 5.6
Restraint violation energy (kJ mol^−1^)	1,466.9 ± 58.1	1,716.1 ± 134.4
Buried surface area	3,171.2 ± 292.7	3,096.8 ± 191.6
*Z*-score	−2.4	−2.2
Binding affinity (ΔG)	−13.4 kcal/mol	−16.4 kcal/mol
Dissociation constant (Kd)	1.4 × 10^−10^ M	8.7 x10^−13^ M

^*a*^ Data represent mean ± standard deviation values obtained from HADDOCK docking analysis, including interaction energies, structural stability metrics, binding affinity (ΔG), and dissociation constant (Kd).

### Molecular dynamics simulation

**Fig 7:**
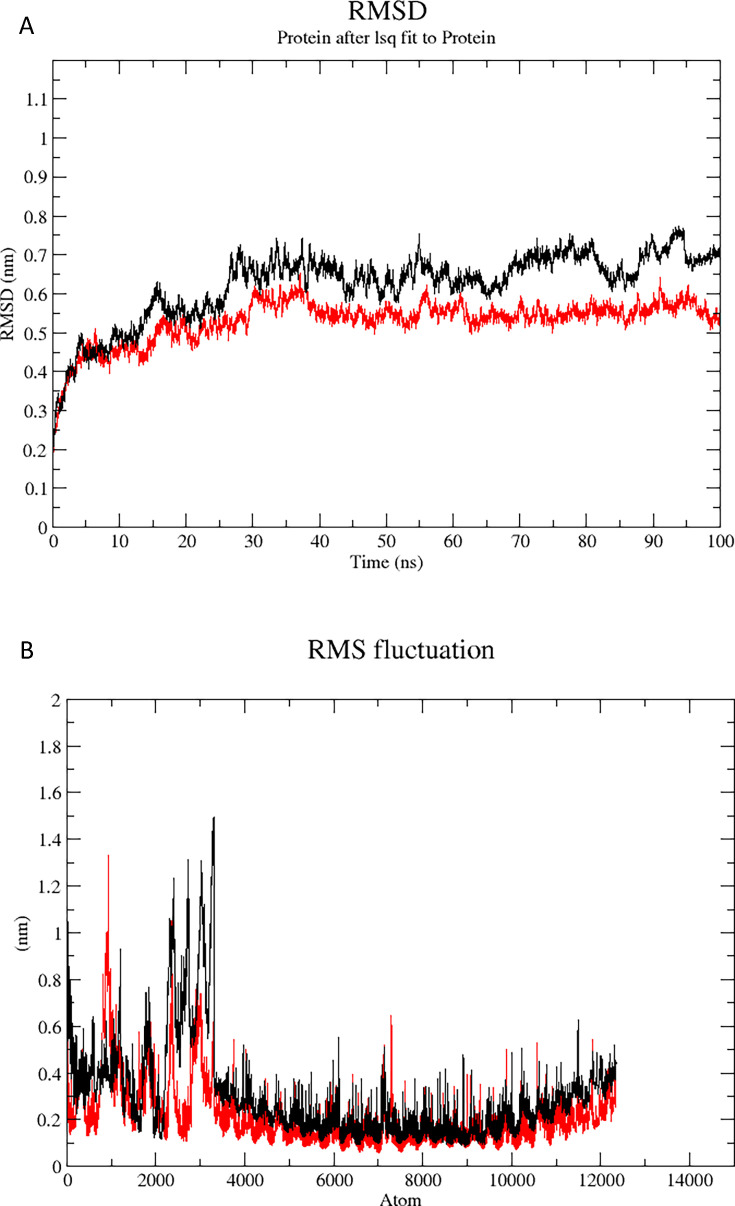
(**A**) RMSD plot showing structural stability of mutant (black) and original (red) vaccine complexes over 100 ns simulation. (**B**) RMSF plot depicting residue flexibility of mutant (black) and original (red) vaccine complexes during simulation.

RMSD analysis was conducted to assess the structural stability and conformational flexibility of the protein complexes during MD simulations. RMSD profiles were plotted over the simulation timescale to monitor conformational shifts and to compare the relative stability of the native and mutant vaccine–TLR4 complexes. A slight elevation in RMSD values, relative to the ideal range, in the mutant vaccine–TLR4 complex was observed. However, this deviation was primarily localized to the flexible loop regions and is not expected to affect the functional integrity of the vaccine construct. Notably, the RMSD trajectory indicated that the mutant vaccine–TLR4 complex maintained comparable conformational stability with respect to the native complex. During the initial 40 ns, a progressive increase in RMSD was observed from approximately 0.3 to 0.7 nm corresponding to structural equilibration. Subsequently, the system stabilized, with RMSD values fluctuating within a narrow range of 0.7 ± 0.05 nm for the remaining 100 ns, indicating a well-equilibrated and stable complex. (Fig. 7A). RMSF analysis was performed to evaluate residue-level flexibility within the protein structure during MD simulations. RMSF quantifies the average positional deviation of each atom from its mean over the simulation trajectory, thereby highlighting regions of structural flexibility. The RMSF profile revealed pronounced fluctuations in residues corresponding to atom indices 2,000–3,000, indicating localized flexibility in this region. In contrast, the remainder of the structure exhibited minimal fluctuations, reflecting overall conformational stability (Fig. 7B).

### Codon optimization and *in silico* cloning

**Fig 8:**
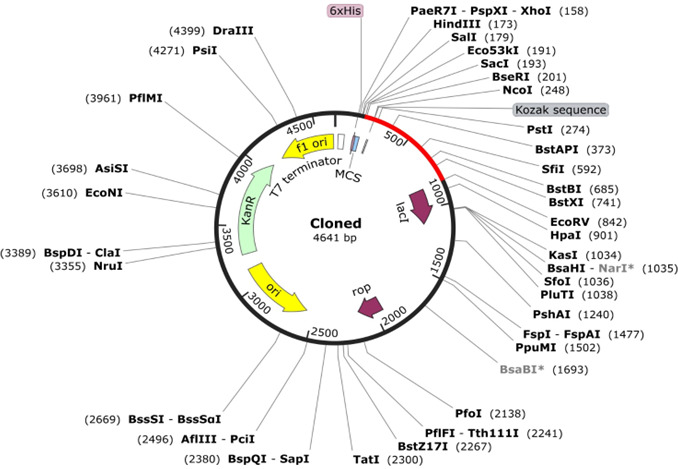
*In silico* restriction cloning of the codon-optimized vaccine gene (red) into the pET-28a(+) expression vector, with the black circle representing the vector backbone.

Due to host-specific codon usage biases that can impede the efficient translation of heterologous genes, codon optimization is a critical strategy to improve expression in a selected host. Codon adaptation for optimal expression in *E. coli* K12 was achieved through the JCat. The optimized sequence achieved a codon adaptation index of 0.602 and a GC content of 64.23%, inferring favorable potential for expression in the *E. coli* system. As illustrated in Fig. 8, the optimized gene sequence was inserted between the Eco53kI and EcoRV restriction sites within the *E. coli* expression vector pET28a(+), resulting in a recombinant clone with a total size of 4,641 base pairs.

### Immune simulation

**Fig 9:**
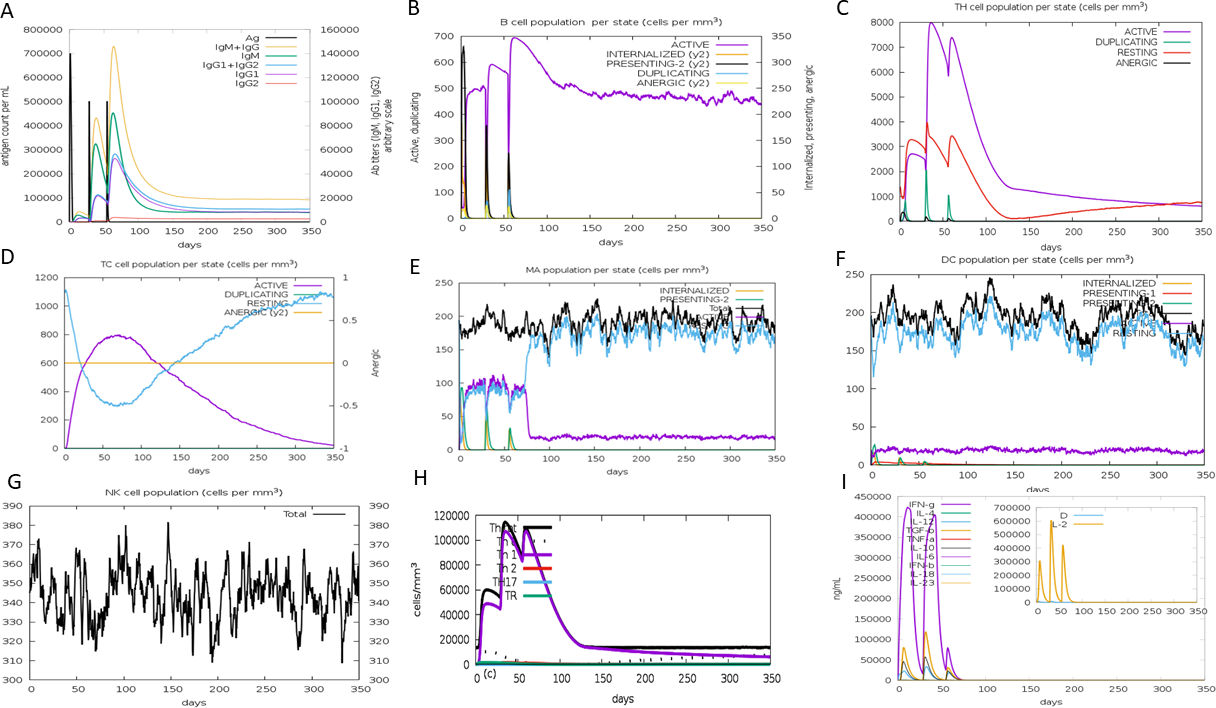
*In silico* simulation of immune responses following three successive vaccine doses. (**A**) Antigen levels and immunoglobulin subtypes, highlighting IgG-mediated primary, secondary, and tertiary responses. (**B**) B-cell states. (**C**) T-helper cells. (**D**) Cytotoxic T cells. (**E**) Macrophages. (**F**) Dynamics of DC population per state over the simulation period, showing active antigen presentation and immune activation. (**G**) Temporal variations in cytokine levels and interleukin concentrations, reflecting immune signaling events following vaccination. (**H**) Kinetics of T-helper cell populations (TH1, TH2, and TH17) and regulatory T cells (Treg) over 350 days, indicating the magnitude and duration of cellular immune responses induced by the vaccine, (**I**) Cytokine and interleukin concentrations across response stages.

The immunogenic potential of the multi-epitope vaccine construct was evaluated by the C-ImmSim server over a simulated period of 350 days. The immune response dynamics revealed a sustained and rigorous activation of both cellular and humoral immunity. Following the first dose, a slight increase in antibody titers, B cells, plasma cells, and helper T (TH) cells was observed, with significant amplification after the second and third doses. Peak IgM + IgG levels reached approximately 140,000 following the third dose administered around day 50 (Fig. 9A). Peak populations of active B cells (~700 cells/mm³) (Fig. 9B), TH cells (~8,000 cells/mm³) (Fig. 9C), and cytotoxic T (TC) cells (~800 cells/mm³) (Fig. 9D) were also recorded, indicating effective induction of both arms of the adaptive immune system. Memory B, TH, and TC cells showed progressive expansion after each dose, persisting through the end of the simulation, suggestive of long-term immunological memory. Additionally, innate immune components, including epithelial cells, macrophages (Fig. 9E), dendritic cells (Fig. 9F), and natural killer (NK) cells (Fig. 9G), were actively recruited, and Th1-dominated response was prominent (Fig. 9H). The vaccine elicited strong cytokine responses—notably, IL-2, IL-10, TGF-β, and IFN-γ—with IFN-γ levels surpassing 400,000 ng/mL (Fig. 9I). Overall, the simulation suggests that the vaccine is capable of inducing a strong Th1 immune response.

## DISCUSSION

Vaccination remains a cornerstone of infectious disease control. However, the development of effective vaccines against gram-negative pathogens such as *A. baumannii* and *P. aeruginosa* continues to present significant challenges (57). These pathogens exhibit extensive intrinsic and acquired resistance mechanisms, resilient biofilm-forming capacity, and remarkable persistence in hospital settings (58). Such parameters not only complicate treatment regimens but also facilitate nosocomial transmission (59). Consequently, both organisms have been classified by the World Health Organization as “critical-priority” pathogens, emphasizing the urgent need for effective preventive strategies, including vaccines (60). Classical vaccine strategies against *A. baumannii* and *P. aeruginosa* have predominantly relied on full-length antigens or limited epitope repertoires derived from virulent antigens, which primarily elicit humoral immune responses. In contrast, immunoinformatics-based vaccine designs, including the present study, employ rational epitope selection to integrate CTL, HTL, and B-cell epitopes derived from conserved protein regions with predicted high HLA-binding affinity and immunogenicity. This epitope-level refinement enables broader population coverage, balanced cellular and humoral immune activation, and reduced susceptibility to antigenic variability, thereby addressing key limitations associated with classical whole-protein or subunit vaccine approaches. Recent vaccine studies against *A. baumannii* have reported promising protective efficacy using protein-based and immunomodulatory vaccine platforms, underscoring the pathogen’s vulnerability to antigen-specific immune targeting (7, 9). Nevertheless, these approaches often rely on limited antigenic repertoires and may be susceptible to immune escape driven by sequence variability. Notably, the IC43 vaccine, comprising recombinant OprF and OprI from *P. aeruginosa*, reached phase II/phase III clinical trials but failed to confer sufficient protection (61). Likewise, for *A. baumannii*, promising candidates such as OmpA and Bap have shown preclinical promise but have yet to reach clinical evaluation (62). These limitations highlight the complexity of designing vaccines capable of overcoming high antigenic variability and immune evasion in gram-negative bacteria (63). To overcome these challenges, we adopted a comprehensive, mutation-aware multi-epitope vaccine design strategy integrating comparative proteomics, subtractive filtering, and advanced immunoinformatics tools. Pan-genome analysis of 30 clinical isolates identified 4,503 conserved proteins, of which 54 were predicted to be extracellular. Following antigenicity and homology screening, three non-toxic, non-homologous, and potentially antigenic proteins were shortlisted as core vaccine targets. From these, we predicted and evaluated CTL, HTL, and B-cell epitopes. After rigorous filtering for antigenicity, allergenicity, toxicity, and MHC-binding affinity, a total of one CTL, six HTL, and three B-cell epitopes were incorporated into the final construct. A distinctive feature of our approach was the inclusion of mutation probability profiling during epitope selection. Residue-level structural analysis using Aggrescan3D identified serine residues within epitopes prone to mutation. Conservative substitutions (e.g., Ser to Thr) were introduced where energetically favorable, improving conformational stability and HLA binding without compromising immunogenicity. This mutation-aware refinement ensures greater evolutionary resilience of the vaccine, mitigating the risk of immune escape, an increasingly important consideration in bacterial vaccine development. The multi-epitope construct, adjuvanted with human β-defensin-3 and joined by cleavable, immunologically inert linkers (EAAAK, AAY, GPGPG, and KK), consisted of 219 amino acids with favorable physicochemical properties: a molecular weight of 23.3 kDa, theoretical pI of 9.55, instability index of 36.3, aliphatic index of 71.28, and a GRAVY score of –0.306, suggesting moderate thermostability and hydrophilicity. These properties collectively indicate a construct with high expression potential and structural integrity. Tertiary structure prediction and refinement yielded a model with 95.8% of residues in favored regions of the Ramachandran plot. Structural quality was further validated by a ProSA *Z*-score of –3.41 and an ERRAT score of 65.93, confirming model reliability. Conformational B-cell epitope analysis predicted surface-accessible regions capable of eliciting strong humoral responses. Protein–protein docking using HADDOCK 2.4 discovered a stable association between the vaccine construct and human TLR4. The complex formed 5 salt bridges, 11 hydrogen bonds, and over 140 non-bonded interactions, with interface areas of 1,683 and 1,458 Å² for vaccine and TLR4, respectively. Binding affinity analysis via PRODIGY indicated a ΔG of –16.4 kcal/mol and a dissociation constant (Kd) of 8.7 × 10⁻¹³ M, substantially more stable than the original protein complex (ΔG = –13.4 kcal/mol). These results underscore the vaccine’s ability to activate innate immune receptors and trigger downstream signaling cascades. Molecular dynamics simulations validated the structural stability and energetically favorable interaction of the vaccine–TLR4 complex. The mutant protein displayed a marginally higher RMSD than the wild-type but remained within the biologically acceptable range (<1.0 nm). RMSD stabilization after 40 ns indicated equilibrium, with the increase likely reflecting localized flexibility rather than global instability. Overall, the mutant retained structural stability throughout the simulation (64). Population coverage analysis indicated 79% global coverage with a PC90 of 0.47, suggesting broad HLA recognition. *In silico* codon adaptation and cloning supported high-level translational expression in *E. coli*. Immune simulations predicted strong and durable responses upon three-dose administration. Elevated IgM and IgG titers, persistent memory B- and T-cell populations, and vigorous cytokine secretion, especially IFN-γ (>400,000 ng/mL), thus, demonstrated skewed Th1 responses and long-term immunological memory. Notably, the vaccine also elicited active recruitment of innate immune components, including macrophages, NK cells, dendritic cells, and epithelial cells, indicating a holistic immunostimulatory profile. Overall, the designed vaccine shows promise for broad protection and requires experimental validation.

### Conclusion

In this study, we present a rationally designed, mutation-aware multi-epitope vaccine construct targeting two WHO-designated critical-priority gram-negative pathogens, *A. baumannii* and *P. aeruginosa*, by integrating pan-genome-based subtractive proteomics with advanced immunoinformatics and structural refinement tools. We identified conserved, surface-exposed antigens and designed a construct comprising CTL, HTL, and B-cell epitopes optimized for immunogenicity, population coverage, and evolutionary stability. Structural modeling and docking analyses confirmed the stability and high-affinity interaction of the vaccine with TLR4, while molecular dynamics simulations supported its conformational resilience. Immune simulations predicted humoral and cellular responses, including long-lived memory populations and skewed Th1 immunity. The vaccine also demonstrated broad HLA population coverage and high expression potential in *E. coli*. Taken together, our results highlight the potential of this multi-epitope construct as a promising candidate for combating polymicrobial infections caused by *A. baumannii* and *P. aeruginosa*. Experimental validation will be critical to assess its protective efficacy *in vivo*.
